# Relationship between nutrition status and muscle mass and its impact on 1-year mortality in patients with aortic stenosis undergoing transcatheter aortic valve implantation

**DOI:** 10.1016/j.ijcrp.2026.200573

**Published:** 2026-01-07

**Authors:** Hiroyo Miyata, Koichiro Matsumura, Kazue Hamamura, Masakazu Yasuda, Shohei Hakozaki, Kyohei Onishi, Eijiro Yagi, Kosuke Fujita, Katsumi Kajihara, Teruyoshi Amagai, Masafumi Ueno, Gaku Nakazawa

**Affiliations:** a Department of Clinical Nutrition, Kindai University Hospital, Osakasayama, Japan; b Department of Cardiology, Kindai University Faculty of Medicine, Osakasayama, Japan; c Department of Rehabilitation, Kindai University Hospital, Osakasayama, Japan; d Department of Cardiology, Sakurabashi Watanabe Mirai Medical Hospital, Osaka, Japan; e Faculty of Health Care Sciences, Department of Clinical Engineering, Jikei University of Health Care Sciences, Osaka, Japan

**Keywords:** Malnutrition, Muscle wasting, Psoas muscle volume index, Aortic stenosis, Transcatheter aortic valve implantation

## Abstract

**Background:**

We aimed to evaluate whether dual assessment of nutritional status and muscle mass could enhance the prediction of 1-year mortality in patients undergoing transcatheter aortic valve implantation (TAVI).

**Methods:**

This retrospective study included 312 consecutive patients who underwent TAVI for aortic stenosis. Nutritional status was determined by calculating the geriatric nutritional risk index (GNRI), with a cutoff of 98. Muscle mass was calculated from the psoas muscle volume index (PMVI). Patients were stratified into three groups based on GNRI and PMVI: Low, middle, and high groups (GNRI: < 98, < 98, and > 98; and below, or below, and above the sex-specific median PMVI, respectively). The primary endpoint was all-cause mortality within 1-year.

**Results:**

After exclusion, 259 patients were included in the analysis, and 22 died within 1-year. Kaplan-Meier survival curves showed a significant difference in all-cause mortality within 1-year among the three groups. Multivariate analysis of the Cox proportional hazards model for factors related to all-cause mortality within 1-year showed no significant association in the middle group when the high group was used as a reference, and the low group was independently associated.

**Conclusion:**

The combined assessment of nutritional status and muscle mass improves the identification of high-risk patients undergoing transcatheter aortic valve implantation and enhances the prognostic accuracy beyond individual markers. This approach may aid in refining the risk stratification and tailoring the perioperative management of patients undergoing TAVI.

## 1 Introduction

The incidence of aortic stenosis has increased significantly with the aging of the population [1]. Transcatheter aortic valve implantation (TAVI) is an established alternative to surgical aortic valve replacement for patients with severe aortic stenosis [2,3]. TAVI is increasingly recognized as the standard treatment in older patients with severe aortic stenosis [1]. The safety and short-term efficacy of TAVI in older patients have been demonstrated in numerous clinical studies. However, the evaluation and stratification of factors influencing long-term postoperative outcomes remain a concern [4].

Many patients undergoing TAVI have a combination of geriatric syndromes such as malnutrition and sarcopenia in addition to the physiological reserve associated with advanced age, and it is becoming increasingly clear that these components affect postoperative recovery and long-term prognosis [5,6]. In particular, nutritional status and reduced skeletal muscle mass are considered pivotal components of physical frailty and are recognized as important prognostic components in patients with cardiovascular disease [7]. Nutritional status and skeletal muscle mass are closely related, and poor nutritional status leads to long-term loss of muscle mass and ultimately to debilitating general conditions such as weight loss and cachexia [8]. However, studies simultaneously assessing multiple components of geriatric syndromes in patients undergoing TAVI and correlating them with prognosis are still limited [4].

The Geriatric Nutritional Risk Index (GNRI), which is calculated based on serum albumin levels and body weight, is widely used as a simple score to assess nutritional risk in older populations. A previous study reported a higher mortality rate in patients with a lower GNRI, chronic heart failure, atrial fibrillation, and even in patients undergoing TAVI, indicating its prognostic usefulness [9]. The psoas muscle mass can be quantitatively evaluated using computed tomography (CT) images and is a reliable indicator of muscle mass, particularly in the trunk of the body. Psoas muscle mass has been used as an indicator of sarcopenia in surgical patients and those undergoing TAVI, with low values being associated with longer hospital stays and increased mortality [10]. Nutritional status (GNRI) and muscle mass (psoas muscle mass) are considered to be important components that independently influence prognosis after TAVI. The combined evaluation of these components is important to achieve more accurate risk stratification and improve the quality of personalised medical care after TAVI. Accordingly, we evaluated a composite of two components, the GNRI and psoas muscle mass, in patients undergoing TAVI, and examined their association with long-term prognosis. The purpose of this study was to provide a comprehensive view of geriatric syndromes based on different aspects of nutritional status and muscle mass and to contribute to the construction of a new stratification index for predicting prognosis after TAVI.

## 2 Methods

### 2.1 Study population

This single-centre retrospective observational study included 312 consecutive patients who underwent TAVI for aortic stenosis between February 2017 and February 2021. Patients were excluded based on the following criteria: in-hospital death (n = 10), maintenance dialysis (n = 9), protein-restricted diet (n = 24), absence of oral intake at preoperative period (n = 5), or loss to follow-up (n = 5). Patients were stratified into three groups using a GNRI cutoff of 98 and the sex-specific median PMVI: GNRI <98 and below the sex-specific median PMVI (low group); GNRI <98 or below the sex-specific median PMVI (middle group); GNRI ≥98 and above the sex-specific median PMVI (high group) (Supplementary Fig. S1). The study protocol was approved by the Ethics Committee of our institute, and patients were enrolled using the opt-out method. The study complied with the principles outlined in the Declaration of Helsinki (1975).

### 2.2 Data collection

Demographic data, including age, sex, body mass index (BMI), blood pressure, New York Heart Association (NYHA) class, comorbidities, and medications at admission, were obtained from hospital records. Pre-procedural laboratory data and echocardiographic parameters were also collected. Physical therapists assessed the Clinical Frailty Scale scores before TAVI. The preoperative Society of Thoracic Surgeons (STS) score was calculated using an online calculator [11]. Procedural details, including access route and duration, were obtained from the surgical records.

### 2.3 Assessment of the GNRI

The GNRI was calculated using preoperative serum albumin levels and BMI at admission using the following formula: GNRI = 14.89 × serum albumin (g/dL) + 41.7 × body mass index/22 [12]. Based on previous literature, a GNRI <98 was used as the cutoff to define malnutrition [9].

### 2.4 Calculation of psoas muscle volume index

The psoas muscle volume index (PMVI) was calculated using preoperative thoracoabdominal CT scans. The analysis was performed using SYNAPSE VINCENT version 5.5 (FUJIFILM Medical Systems, Tokyo, Japan) (Supplementary Fig. S2). The volume of the psoas muscle was determined using automated segmentation. The volumes of the bilateral psoas muscles were automatically calculated using the built-in computation tool in the Vincent software. The PMVI was calculated by normalising the total psoas muscle volume (cm^3^) to the body surface area (m^2^). All measurements were performed by independent observers who were blinded to the clinical data. Previous studies using SYNAPSE VINCENT have reported excellent interobserver reproducibility for automatically measured psoas muscle volumes, with intraclass correlation coefficients of >0.99, indicating that the measurement error because of different observers is negligible [13].

### 2.5 Outcome measures

The primary outcome was all-cause mortality within 1-year. Mortality data were obtained from hospital records or responses to questionnaires sent to institutions that followed the patients. The secondary outcomes and 1-year all-cause mortality were compared between groups classified by a GNRI cutoff of 98 and between groups classified by the sex-specific median PMVI.

### 2.6 Statistical analysis

Continuous variables are expressed as medians with interquartile ranges and were compared using the Kruskal–Wallis H test. Categorical variables are expressed as percentages and were compared using the chi-square test. Kaplan–Meier survival analysis was used to estimate event-free survival, and differences between groups were evaluated using the log-rank test. Multivariate analysis was performed using the Cox proportional hazards model, adjusting for age, sex, B-type natriuretic peptide level, left ventricular ejection fraction, and STS score >8. All statistical analyses were conducted using SPSS (version 23.0; IBM Corporation, Armonk, NY, USA), and a p-value <0.05 was considered statistically significant.

## 3 Results

### 3.1 Patient characteristics

**Fig. 1:**
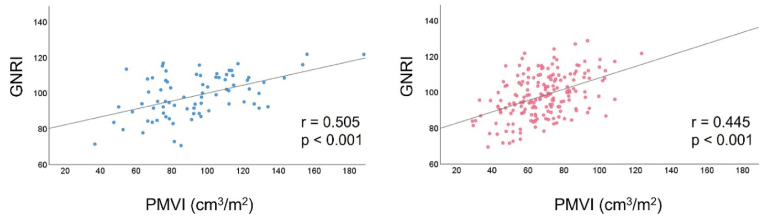
Relationship between the GNRI and sex-specific PMVI. GNRI: Geriatric Nutritional Risk Index, PMVI: Psoas Muscle Volume Index.

Among the 259 patients (median age, 85 years; 70 % female), 8.5 % (22 patients) died within 1-year Fig. 1 shows the correlation between the GNRI and PMVI according to sex. The results revealed a significant correlation between the GNRI and PMVI in both sexes (male: r = 0.505, p < 0.001; female: r = 0.445, p < 0.001). The median PMVI was 93.2 cm^3^/m^2^ for males and 68.0 cm^3^/m^2^ for females. Patients were stratified into three groups by a GNRI cutoff of 98 and the sex-specific median PMVI: GNRI <98 and below the sex-specific median PMVI (low group, n = 86); GNRI <98 or below the sex-specific median PMVI (middle group, n = 85); GNRI ≥98 and above the sex-specific median PMVI (high group, n = 88) (Supplemental Fig. 1). The results revealed significant differences in age, BMI, NYHA class, dyslipidaemia, and history of hospitalization for heart failure between the three groups at the time of admission (Table 1). The rates of female sex, atrial fibrillation, and history of myocardial infarction were similar among the three groups. The proportion of patients taking loop diuretics was significantly different among the three groups, whereas the proportions of patients taking angiotensin-converting enzyme inhibitors/angiotensin II receptor blockers and beta-blockers were similar. Preoperative laboratory data showed significant differences in haemoglobin, serum albumin, high-sensitivity C-reactive protein, and B-type natriuretic peptide levels among the three groups. Echocardiography revealed significant differences in left ventricular ejection fraction and aortic valve area.

**Table 1:** Patient characteristics at admission.

	Low group (n = 86)	Middle group (n = 85)	High group (n = 88)	*p*–value
Age, years	86 [83–90]	86 [82–89]	84 [79–86]	<0.01
Female	60 (70)	63 (74)	59 (67)	0.59
BMI, kg/m^2^	19.1 [17.2–20.8]	23.1 [20.6–25.2]	24.4 [22.9–27.1]	<0.01
Systolic blood pressure, mm Hg	122 [106–140]	126 [113–138]	128 [115–136]	0.55
NYHA class III/IV	41 (48)	25 (29)	22 (25)	<0.01
Comorbidity				
Hypertension	68 (79)	67 (79)	72 (82)	0.86
Dyslipidaemia	37 (43)	52 (61)	65 (74)	<0.01
Diabetes mellitus	18 (21)	22 (26)	34 (39)	0.03
Atrial fibrillation	24 (28)	21 (25)	20 (23)	0.73
COPD	8 (9)	2 (2)	7 (8)	0.15
Prior heart failure hospitalization	55 (64)	39 (46)	31 (35)	<0.01
Prior myocardial infarction	5 (6)	6 (7)	9 (10)	0.53
Prescription				
Loop diuretics	45 (52)	36 (42)	28 (32)	0.02
ACE-I/ARB	44 (51)	48 (57)	54 (61)	0.40
Beta-blocker	20 (23)	22 (26)	22 (25)	0.92
Laboratory				
Haemoglobin, g/dL	10.8 [9.9–12.0]	11.4 [10.0–12.8]	12.4 [11.4–13.5]	<0.01
Serum creatinine, mg/dL	0.95 [0.69–1.25]	0.93 [0.70–1.17]	0.85 [0.74–1.03]	0.33
Serum albumin, g/dL	3.5 [3.2–3.8]	3.8 [3.4–4.1]	4.0 [3.8–4.2]	<0.01
High-sensitive CRP, mg/dL	0.19 [0.05–0.59]	0.11 [0.04–0.29]	0.08 [0.03–0.22]	<0.01
BNP, pg/mL	367 [171–633]	180 [92–385]	142 [66–297]	<0.01
LVEF, %	64 [56–70]	69 [63–73]	67 [61–71]	0.01

Data are presented as medians (25th–75th percentiles) or numbers (%).

ACE-I/ARB: Angiotensin–converting enzyme inhibitor/angiotensin II receptor blocker, BNP: B-type natriuretic peptide, COPD: Chronic obstructive pulmonary disease, CRP: C-reactive protein, LVEF: Left ventricular ejection fraction, MRA: Mineralocorticoid receptor antagonist, NYHA: New York Heart Association.

The results showed significant differences in the clinical frailty score, STS score, and percentage of emergency procedures between the three groups (Table 2), while no significant differences were observed in the approach site or operative time for TAVI.

**Table 2:** Procedural characteristics.

	Low group (n = 86)	Middle group (n = 85)	High group (n = 88)	*p*–value
Clinical frailty score	4 [4–5]	4 [4–5]	4 [3–4]	<0.01
STS score	7 [5–10]	6 [4–9]	5 [3–6]	<0.01
STS score >8	31 (36)	25 (29)	11 (13)	<0.01
Emergency	21 (24)	13 (15)	4 (5)	<0.01
Approach site				0.31
Transfemoral	78 (91)	82 (97)	82 (93)	
Transapical	3 (4)	2 (2)	2 (2)	
Transaortic	0 (0)	1 (1)	0 (0)	
Transsubclavian	5 (6)	0 (0)	4 (5)	
Operation time, min	61 [47–81]	56 [45–71]	57 [43–73]	0.26
GNRI	89 [83–93]	98 [91–107]	108 [102–113]	<0.01
GNRI <98	86 (100)	42 (49)	0 (0)	<0.01
PMVI, cm^3^/m^2^	57.9 [49.0–66.6]	72.6 [60.2–80.0]	89.9 [74.8–105.1]	<0.01
PMVI in males, cm^3^/m^2^	75.3 [63.7–81.5]	83.2 [74.6–103.7]	112.1 [102.6–123.5]	<0.01
PMVI in females, cm^3^/m^2^	55.8 [44.8–62.0]	68.7 [59.5–76.9]	81.8 [72.8–91.3]	<0.01

Data are presented as medians (25th–75th percentiles) or numbers (%).

GNRI: Geriatric Nutritional Risk Index, PMVI: Psoas Muscle Volume Index, STS: Society of Thoracic Surgeons.

### 3.2 Primary outcome

**Fig. 2:**
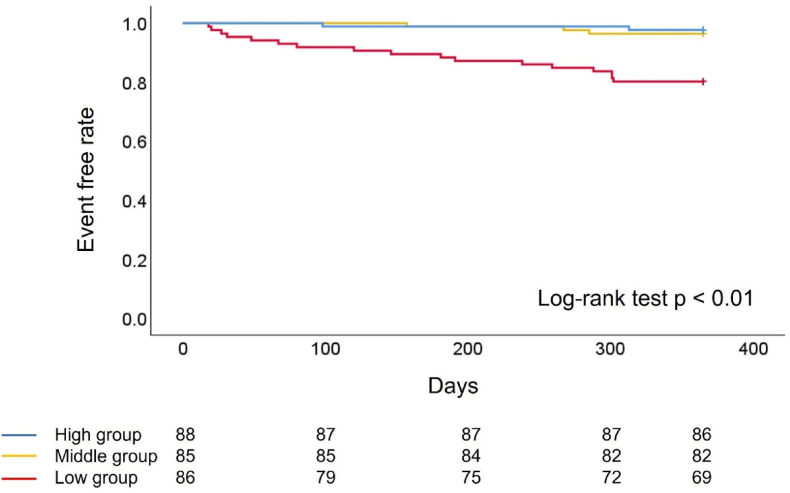
Kaplan–Meier analysis of patients who were free of all-cause death. Low group (GNRI <98 and below the sex-specific median PMVI), middle group (GNRI <98 or below the sex-specific median PMVI), and high group (GNRI ≥98 and above the sex-specific median PMVI). GNRI: Geriatric Nutritional Risk Index, PMVI: Psoas Muscle Volume Index.

Kaplan–Meier survival curve analysis showed a significant difference in 1-year mortality among the three groups: low group, 19.8 % (17 of 86 patients); middle group, 3.5 % (3 of 85 patients); and high group, 2.3 % (2 of 88 patients); log-rank test p < 0.01 (Fig. 2). Multivariate analysis of the Cox proportional hazards model for 1-year mortality, adjusted for age, sex, B-type natriuretic peptide level, left ventricular ejection fraction, and STS score >8, showed no significant difference between the high and middle groups (hazard ratio [HR]: 1.51, 95 % confidence interval [CI]: 0.24–9.66, p = 0.66), but did show a significant increase in the low group (HR: 8.43, 95 % CI: 1.75–40.52, p < 0.01) (Table 3).

**Table 3:** Cox regression hazard model for the all-cause death.

	Unadjusted HR (95 % CI)	*p*-value	Adjusted HR^a^ (95 % CI)	*p*-value
High group (GNRI ≥98 and PMVI ≥ the sex-specific median)	Reference		Reference	
Middle group (GNRI <98 or PMVI<the sex-specific median)	1.60 (0.27–9.57)	0.61	1.52 (0.24–9.74)	0.66
Low group (GNRI <98 and PMVI<the sex-specific median)	9.78 (2.26–42.33)	<0.01	8.46 (1.76–40.62)	<0.01

BNP: B-type natriuretic peptide, CI: Confidence interval, HR: Hazard ratio, LVEF: Left ventricular ejection fraction, PMVI: Psoas Muscle Volume Index.

a Data on age, female sex, BNP, LVEF, and STS score >8 were collected.

### 3.3 Sub-analysis

When patients were stratified by a GNRI of 98, patients with GNRI <98 had a significantly higher incidence of 1-year mortality than those with a GNRI ≥98 (15.6 % [20 of 128 patients] vs. 1.5 % [2 of 131 patients], log-rank test p < 0.01, Supplementary Fig. S3). Likewise, patients were stratified by sex-specific median PMVI, resulting in patients below the sex-specific median PMVI having a significantly higher incidence of 1-year mortality than those above the sex-specific median PMVI (13.2 % [17 of 129 patients] vs. 3.8 % [5 of 130 patients], log-rank test p < 0.01; Supplementary Fig. S4).

## 4 Discussion

We investigated the association between 1-year all-cause mortality and a combined assessment of two geriatric-related components (preoperative nutritional status and muscle mass) in patients undergoing TAVI. Our results revealed that patients with malnutrition, as assessed by the GNRI, or low muscle mass, as assessed by the sex-specific PMVI, did not show a significant increase in mortality compared to those with preserved status in both components. Patients with malnutrition and low muscle mass exhibited significantly higher mortality rates. Additionally, there was a strong correlation between the GNRI and PMVI. These findings highlight the importance of a comprehensive assessment incorporating multiple geriatric-related components rather than relying on a single prognostic component for risk stratification following TAVI.

Frailty and malnutrition are distinct geriatric-related components but are closely related. In a study by Ishizu et al. assessment using the GNRI showed that 71 % of frail patients were moderately or more malnourished [14]. In contrast, the controlling nutrition status score and the prognostic nutritional index showed less of this overlap, indicating that the extent of overlap between frail and malnourished patients varies depending on the assessment index. Sarcopenia is considered a pivotal component linking frailty and malnutrition [15]. A subanalysis of the FRAILTY-AVR study showed that 21 % of patients undergoing TAVI had sarcopenia, which was associated with an increase in mortality, functional disability, and discharge to nursing homes at 1-year [16]. In addition, 63 % had pre-sarcopenia (loss of either muscle mass or strength), which was also associated with poor prognosis. In our study, we found a strong correlation between the GNRI and PMVI, suggesting a close association between nutritional status and muscle mass.

The GNRI is a widely used screening tool that provides a simple assessment of nutritional risk in the elderly population. It is calculated based on serum albumin levels and body weight and has been reported to correlate with worse prognosis due to poor nutritional status [12,14]. Most older patients undergoing TAVI are at nutritional risk due to chronic disease and frailty, and the GNRI is a useful indicator reflecting these background pathologies. Indeed, patients undergoing TAVI with a low GNRI have been shown to be at a higher risk for postoperative mortality and complications, consistent with our findings [9,14]. However, the GNRI is a component focused on serum albumin levels and does not directly reflect factors such as diet, dietary intake, inflammatory markers, or physical activity [17]. It is also susceptible to chronic inflammation and body water content and may not always reflect pure nutritional status, such as in the case of complications of acute heart failure. Therefore, it is advisable to use multiple geriatric-related components to assess patient prognoses. The GNRI has shown strong prognostic performance in patients with heart failure and those undergoing TAVI, and is frequently used to assess nutritional vulnerability in older adults with cardiovascular disease [14,18]. As the GNRI does not require detailed interviews or dietary assessments, it enables a simple and objective evaluation of malnutrition and risk stratification in routine clinical practice.

The psoas muscle is an important muscle segment that supports trunk stability and is closely involved in activities directly related to daily life, such as standing and sitting, and psoas muscle volume has been used as an objective indicator of geriatric syndromes, such as sarcopenia and frailty [16,19]. Evaluation of the psoas muscle mass can be performed noninvasively and quantitatively using non-contrast CT images before TAVI. Patients with lower psoas muscle volumes have increased in-hospital mortality and rehospitalisation after TAVI, suggesting an association between physical vulnerability and prognosis [10,20]. Evaluation of the psoas muscle mass is clinically important because it is associated with the risk of postoperative complications and delayed recovery. However, the psoas muscle mass is only an assessment of muscle mass and cannot be used to evaluate qualitative aspects such as muscle strength and physical function.

Inflammation often leads to malnutrition and sarcopenia among older adults. Chronic inflammation contributes to appetite loss, suppression of protein anabolism, and accelerated skeletal muscle catabolism, thereby worsening the nutritional status and muscle mass, ultimately leading to a phenotype known as inflammatory sarcopenia [21,22]. Furthermore, malnutrition and reduced muscle mass exacerbate inflammatory responses through impaired immune function and increased oxidative stress, thereby creating a self-perpetuating cycle. The interrelationship among malnutrition, sarcopenia, and inflammation has been reported to be strongly associated with adverse long-term outcomes in a wide spectrum of cardiovascular diseases [23,24]. Increased inflammatory activity promotes cardiac remodelling, endothelial dysfunction, and a prothrombotic state, thereby increasing the risk of cardiovascular events, while simultaneously diminishing physiological resilience. Therefore, in older patients undergoing TAVI, malnutrition and reduced muscle mass may reflect underlying chronic inflammation that contributes to impaired postoperative recovery and poor long-term prognosis.

In our study, patients with low GNRI and PMVI exhibited markedly worse outcomes, indicating that these individuals may represent a phenotype of advanced biological frailty characterised by combined deficits in nutrition, muscle mass, and inflammatory regulation. In contrast, the middle group, defined as having either a low GNRI or low PMVI alone, showed a 1-year mortality rate comparable to that of the group with both parameters preserved. This indicates that aging-related vulnerability in these patients may remain at an early stage. Therefore, even if one domain (nutritional status or muscle mass) is impaired, preservation of the other domain may partially compensate for reductions in physiological reserves, thus preventing clinically evident deterioration in outcomes. This concept of partial frailty is recognized in geriatric medicine, whereas impairment in a single domain alone does not necessarily lead to a clear functional decline or increased clinical events [25,26]. Only when malnutrition and muscle loss progress concurrently do compounded vulnerabilities, such as immune dysfunction, heightened chronic inflammation, and diminished metabolic reserve, become clinically manifested, potentially leading to a worse prognosis.

A notable finding of our study is that combining the two indices, the GNRI as a nutritional index and the PMVI as a muscle mass index, enabled the stratification of the high-risk group. Both assessment parameters used in our study are based on information that is readily obtainable in routine clinical settings. Importantly, they do not require additional testing or specialised equipment, making them feasible and cost- and time-efficient screening tools. From a clinical perspective, the combined evaluation of the GNRI and PMVI may aid in refining the selection criteria for TAVI and optimising postoperative management strategies. Nutritional- and exercise-based therapeutic interventions have been shown to improve nutritional status and muscle mass, and some investigations have reported beneficial effects on long-term clinical outcomes [21,27,28]. These findings indicate that multimodal perioperative interventions may be effective in high-risk TAVI patients with poor nutritional status and sarcopenia. However, this hypothesis requires validation through well-designed prospective interventional trials. Conversely, patients with favourable profiles in both components may derive the greatest benefit from TAVI, with potentially higher procedural safety and efficacy. Future research should aim to advance these assessment strategies from prognostic tools to frameworks for designing individualised interventions.

Our study has several limitations. First, it is a retrospective analysis, which limited our ability to confirm causal relationships. Second, it is a single-centre analysis that requires careful interpretation of external validity. In addition, the relatively limited sample size may have influenced the statistical power to detect differences in long-term outcomes. Finally, we did not assess other geriatric-related factors (such as cognitive function, mental status, and social support) or potential confounders (such as renal dysfunction, inflammation, and anaemia), which may be insufficient for an overall vulnerability assessment. Moreover, we did not examine outcomes beyond one year, and the differences in prognosis among the three groups may vary over a longer follow-up period. Further validation incorporating a comprehensive frailty index and a multicentre prospective study is required.

## 5 Conclusion

We investigated the association between long-term outcomes and two geriatric-related components: the GNRI and PMVI. Our findings demonstrated that a low value in either the GNRI or PMVI alone was not associated with a significantly increased 1-year mortality risk. However, patients with low values for both components exhibited markedly worse outcomes. This suggests that the combined assessment of nutritional status and muscle mass improves the accuracy of prognostic stratification and enables the identification of high-risk patients who may be missed in a single-parameter evaluation. Given that both the GNRI and PMVI can be readily obtained in daily clinical practice without the need for additional tests or equipment, this approach offers high clinical utility for comprehensive risk assessment and the formulation of individualised management strategies before TAVI. Future directions should include prospective multicentre studies and integration of additional geriatric-related factors to further refine the prognostic models.

## CRediT authorship contribution statement

**Hiroyo Miyata:** Writing – original draft, Methodology, Investigation, Formal analysis, Data curation, Conceptualization. **Koichiro Matsumura:** Writing – original draft, Methodology, Formal analysis, Data curation, Conceptualization. **Kazue Hamamura:** Investigation, Formal analysis, Data curation. **Masakazu Yasuda:** Investigation, Formal analysis, Data curation. **Shohei Hakozaki:** Investigation, Formal analysis, Data curation. **Kyohei Onishi:** Investigation, Formal analysis, Data curation. **Eijiro Yagi:** Investigation, Formal analysis, Data curation. **Kosuke Fujita:** Investigation, Formal analysis, Data curation. **Katsumi Kajihara:** Investigation, Formal analysis, Data curation. **Teruyoshi Amagai:** Writing – original draft, Supervision, Methodology, Conceptualization. **Masafumi Ueno:** Writing – original draft, Supervision, Conceptualization. **Gaku Nakazawa:** Writing – original draft, Visualization, Supervision, Conceptualization.

## Data availability statement

The data that support the findings of this study are available from the corresponding author upon reasonable request.

## Funding statement

This research did not receive any specific grant from funding agencies in the public, commercial, or not-for-profit sectors.

## Declaration of competing interest

The authors declare no conflicts of interest.
