# Supplementary material for: Relationship between nutrition status and muscle mass and its impact on 1-year mortality in patients with aortic stenosis undergoing transcatheter aortic valve implantation
Source: Int J Cardiol Cardiovasc Risk Prev. 2026 Jan 7;28:200573. doi: 10.1016/j.ijcrp.2026.200573 (PMC12824908; doi:10.1016/j.ijcrp.2026.200573)
Supplement: Multimedia component 1 [file mmc1.docx]

Relationship between nutrition status and muscle mass and its impact on 1-year mortality in patients with aortic stenosis undergoing transcatheter aortic valve implantation

Supplementary materials


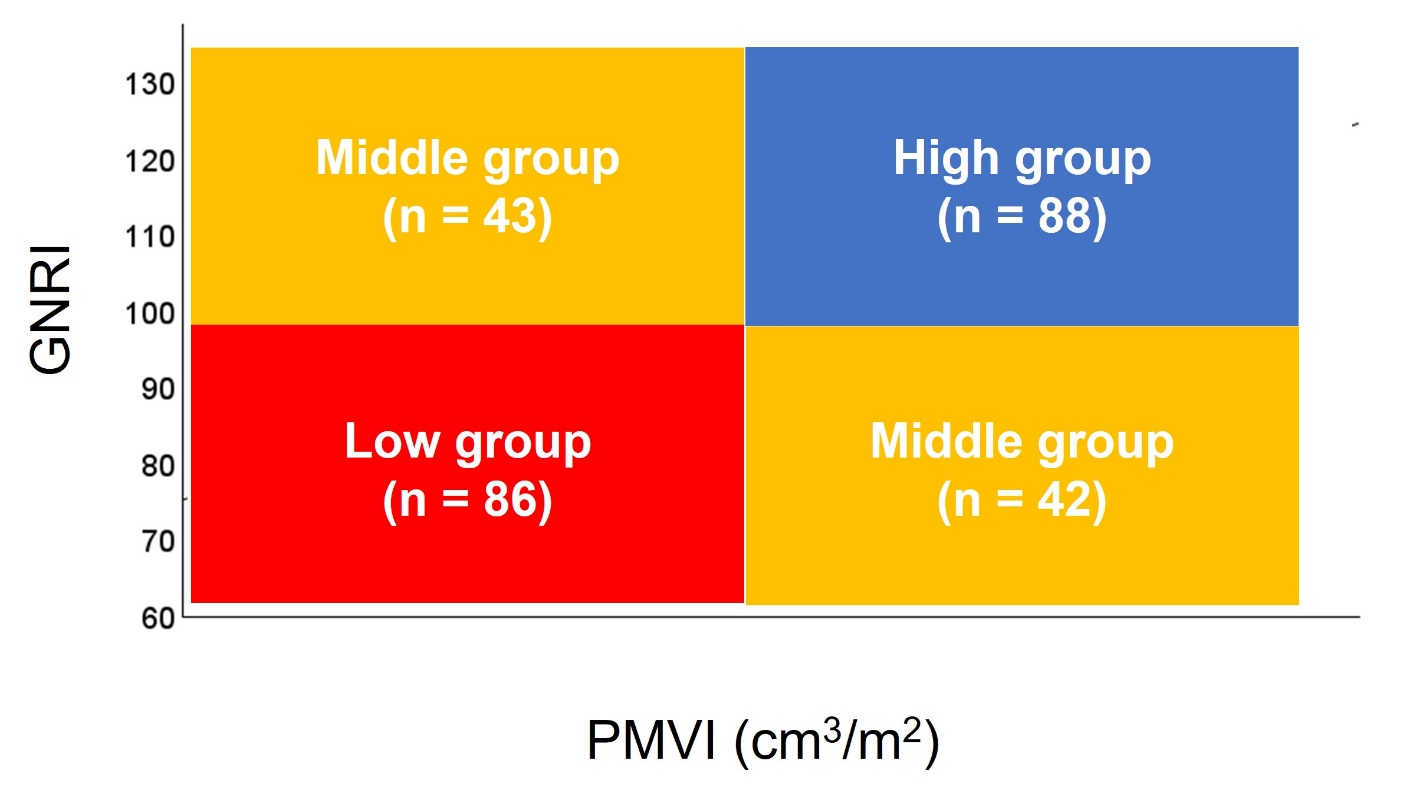


**Supplementary Figure S1. Patients were stratified into three groups based on the GNRI and PMVI**

GNRI: Geriatric nutritional risk index, PMVI: Psoas muscle volume index.


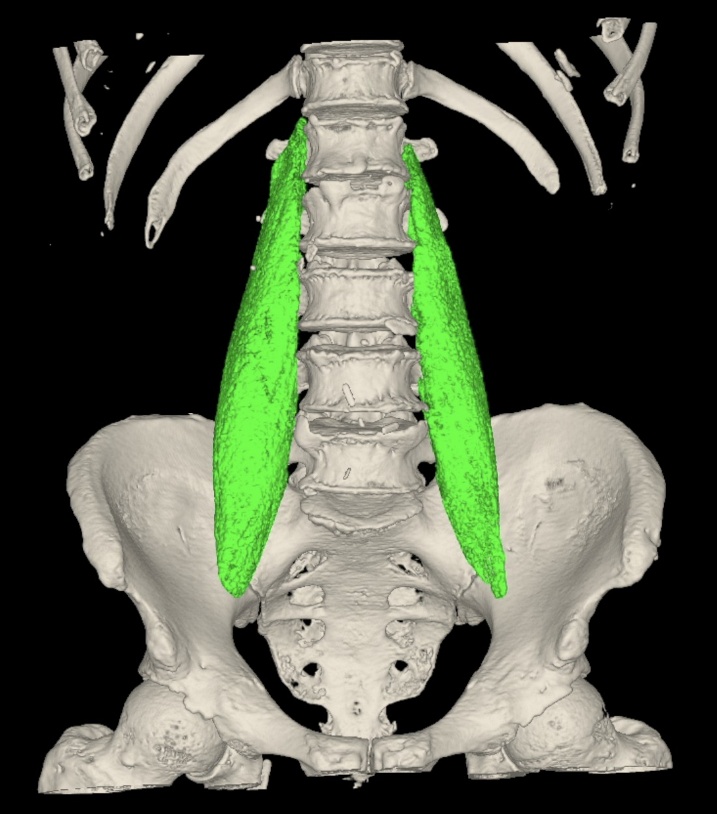


**Supplementary Figure S2. Representative image of psoas muscle volume assessment using preoperative non-contrast thoracoabdominal CT**

CT: computed tomography


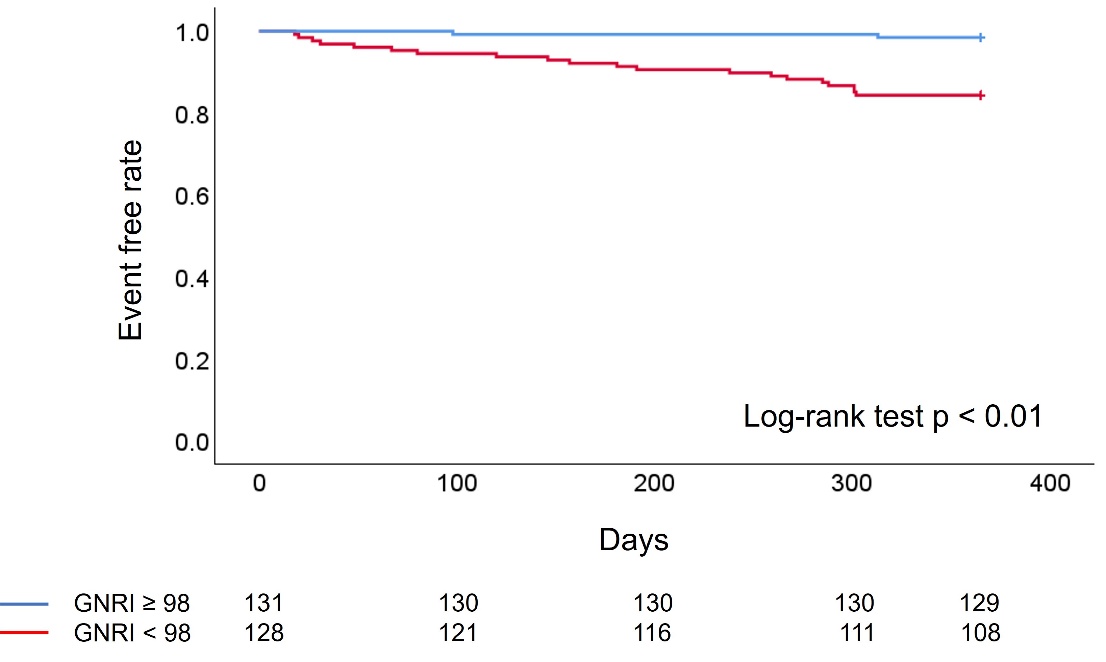


**Supplemental Figure S3. Kaplan–Meier analysis of patients who were free of all-cause death classified by GNRI 98**

GNRI: Geriatric nutritional risk index.

**
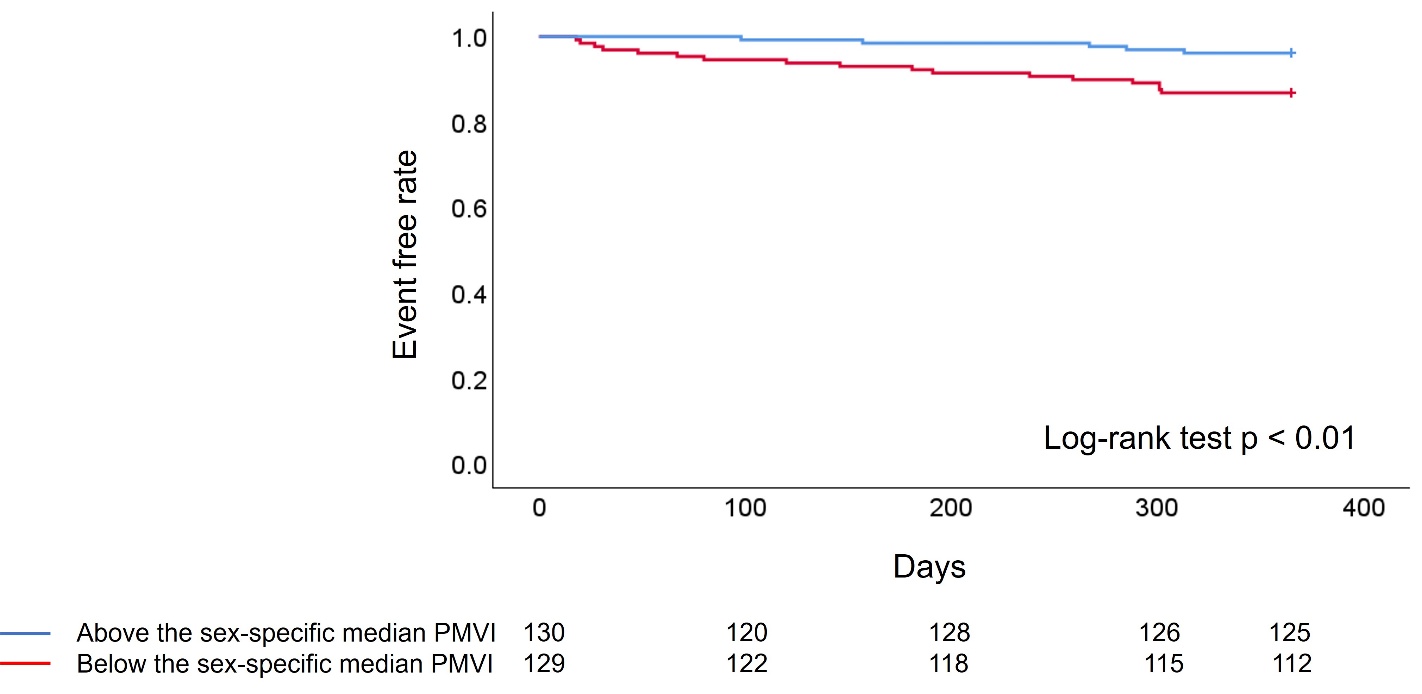
**

**Supplemental Figure S4. Kaplan–Meier analysis of patients who were free of all-cause death classified by sex-specific median PMVI**

PMVI: Psoas muscle volume index.
